# Cardiovascular Autonomic Deficits in Different Types of Achalasia

**DOI:** 10.7759/cureus.59444

**Published:** 2024-05-01

**Authors:** Abhijith Anil, Ritesh K Netam, Atanu Roy, Dinu S Chandran, Ashok Kumar Jaryal, Govind K Makharia, Rajinder Parshad, Kishore K Deepak

**Affiliations:** 1 Department of Physiology, All India Institute of Medical Sciences, New Delhi, New Delhi, IND; 2 Department of Physiology, Institute of Medical Sciences, Banaras Hindu University, Varanasi, IND; 3 Department of Gastroenterology and Human Nutrition, All India Institute of Medical Sciences, New Delhi, New Delhi, IND; 4 Department of Surgical Disciplines, All India Institute of Medical Sciences, New Delhi, New Delhi, IND

**Keywords:** heart rate variability (hrv), lower esophageal sphincter (les), cardiac autonomic function, high resolution esophageal manometry, achalasia cardia

## Abstract

Background and objective

Achalasia cardia is a primary esophageal motility disorder, and the etiopathology of this disease's progression is not known. Moreover, autonomic dysfunction has not been studied in different types of achalasia. In light of this, we aimed to address this lack of data in this study.

Methods

The diagnosis of achalasia was done using high-resolution esophageal manometry (HRM)-based Chicago classification v4.0. Autonomic function tests (AFT) such as the head-up tilt test, deep breathing test (DBT), Valsalva maneuver (VM), handgrip test (HGT), and cold pressor test (CPT), as well as the heart rate variability (HRV) test, were performed among the cohort and the results were compared with those of 39 age- and sex-matched healthy controls.

Results

AFT and HRV tests were done on 62 patients (30 achalasia type I, 28 type II, and 4 type III) and compared with 39 age- and sex-matched healthy controls. The mean duration of symptoms, high Eckardt score, and dysphagia were most common in type I achalasia, followed by type II and III. The results of AFT showed a generalized loss of parasympathetic and baroreflex-independent sympathetic reactivity in all types of achalasia. However, baroreflex-dependent cardiovascular adrenergic reactivity was normal. Regarding cardiac autonomic tone, there was a loss of parasympathetic and sympathetic influence, but sympathovagal balance was maintained. The severity of the loss of autonomic functions was higher in type I, followed by type II.

Conclusions

In all types of achalasia, parasympathetic reactivity, baroreflex-independent sympathetic reactivity, and cardiac autonomic tone were lower compared to healthy controls, and the severity of dysfunction increased during the progression of the disease from type II to type I.

## Introduction

Achalasia is a primary motility disorder of the esophagus, and it is characterized by a loss of relaxation of the lower esophageal sphincter (LES) and the absence of peristalsis in the esophagus [1]. Achalasia is classified into three subtypes based on the Chicago classification: types I, II, and III. Type I achalasia (classic achalasia) is characterized by the absence of esophageal body smooth muscle contractility and no esophageal pressurization. Type II achalasia (the most common type) is characterized by absent peristalsis with abnormal panesophageal high-pressure patterns. Type III achalasia, which is the least common type, is characterized by spastic contraction of the distal esophagus in at least 20% of swallows [2].

The primary pathology in achalasia is the selective loss of inhibitory neurons in the myenteric plexus of the esophageal body and LES [3]. Genetic, viral, inflammatory, autoimmune, and neurodegenerative factors are considered potentially causative of or promoting the disease [4]. Cadaveric and biopsy studies have shown a loss of inhibitory ganglion cells in the myenteric plexus, abnormalities in the vagus nerve similar to Wallerian degeneration, and lesions of the dorsal motor nucleus of the vagus, which is suggestive of neuronal damage from the distal to the proximal end [5,6]. The vagus nerve is also associated with the parasympathetic fibers that supply the heart, esophagus, stomach, pancreas, spleen, adrenal glands, and small intestines [7]. Previous studies have reported the presence of autonomic dysfunction in patients with achalasia [8], but changes occurring in different types of achalasia have not been explored. The study by Salvadore et al. [9] has shown that manometry patterns seen in various types of achalasia could represent different stages in the evolution of the disease, where pattern III progresses to II and finally to I.

The goal of achalasia treatment is to manage the symptoms of dysphagia and related complications by the dilatation of the esophagogastric junction (EGJ). Several treatments can be tailored according to the patient’s overall health status. Pneumatic balloon dilatation (PBD), peroral endoscopic myotomy (POEM), and laparoscopic Heller myotomy (LHM) provide similarly effective long-term results for esophageal achalasia. In patients whose condition is too poor for endoscopic treatment or surgery, botulinum injection or oral medications like calcium channel blockers, nitrates, anticholinergics, phosphodiesterase inhibitors, and ß-adrenergic agonists may be helpful. However, there is no specific therapy targeting the underlying disease process, as the pathogenesis of impaired esophageal peristalsis and poor esophageal sphincter relaxation remains unclear [10]. The present study was designed to assess the extraesophageal autonomic dysfunction associated with disease progression in achalasia. The parasympathetic reactivity, sympathetic reactivity, and cardiac autonomic tone in different types of achalasia were evaluated in achalasia patients and compared with healthy controls to determine and assess the presence of global or systemic autonomic dysfunction.

## Materials and methods

**Figure 1:**
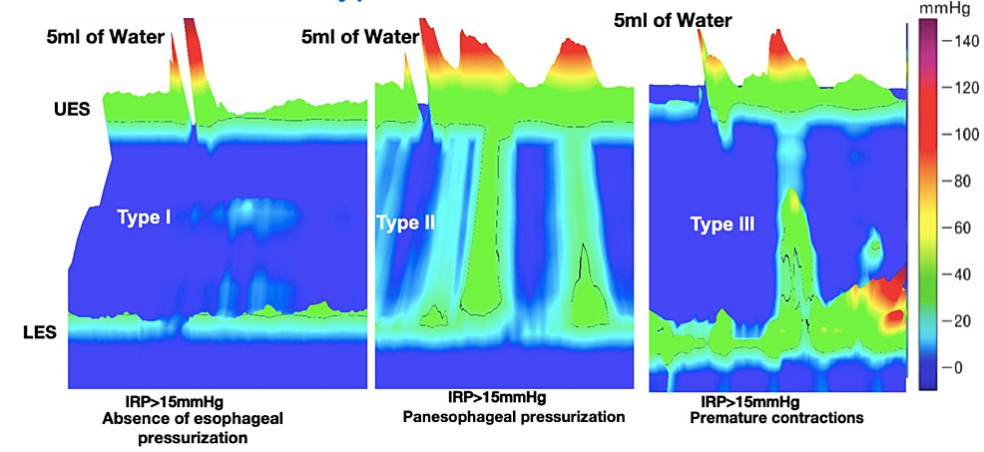
High-resolution manometry (HRM) recordings of the esophagus during a single wet swallow of 5 ml bolus of water in different types of achalasia In type I achalasia, a 100% absence of esophageal pressurization or peristalsis and impaired deglutition inhibition leading to raised integrated relaxation pressure (IRP) of more than 15 mmHg was noted. In type II achalasia, apart from failed peristalsis and IRP of more than 15mmHg, at least 20% of swallows showed panesophageal pressurization or spastic contractions throughout the body of the esophagus. In type III achalasia, apart from failed peristalsis and IRP of more than 15 mmHg, at least 20% of swallows showed premature contractions or spastic contraction in the lower body of the esophagus IRP: integrated relaxation pressure; LES: lower esophageal sphincter; UES: upper esophageal sphincter

**Figure 2:**
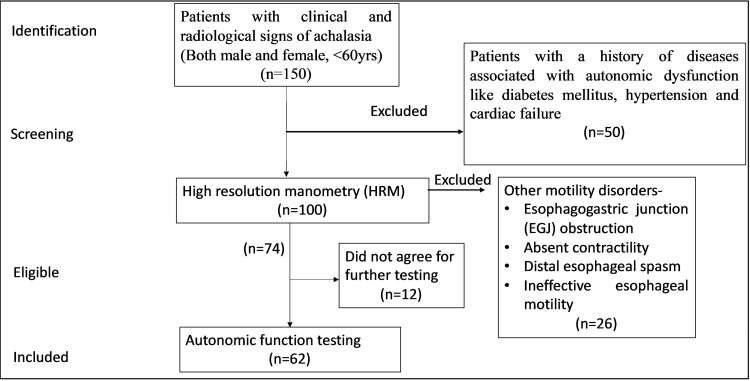
Strengthening the reporting of observational studies in epidemiology (STROBE) flow diagram depicting the inclusion and exclusion criteria

The study was conducted in the Department of Physiology in collaboration with the Department of Gastrointestinal and Human Nutrition and the Department of Surgical Disciplines of the All India Institute of Medical Sciences (AIIMS), New Delhi. The study protocol was approved by the Institute Ethics Committee, (AIIMS) (IECPG-374/30.08.2018). A total of 100 patients with clinical and radiological signs of achalasia were recruited. Both males and females aged less than or equal to 60 years old were recruited. Patients with a history of diseases associated with autonomic dysfunction, such as diabetes mellitus, hypertension, and cardiac failure, were excluded from the study. Diagnosis of different types of achalasia was done using high-resolution manometry, as shown in Figure 1. Then autonomic function testing (AFT) was done. Strengthening the reporting of observational studies in epidemiology (STROBE) flow diagram depicting the inclusion and exclusion criteria is shown in Figure 2.

High-resolution manometry (HRM)

The subjects were instructed to abstain from tea, coffee, and heavy or vigorous exercise (more than 6 metabolic equivalent of task (METS)] for at least 12 hours before the procedure. They were asked to report to the gastrointestinal manometry lab at 9:00 a.m. after an overnight fast (six to eight hours). Reports of barium esophagography and endoscopic examination were inspected to rule out any obstructive pathology and to assess the patency and shape of the esophagus. The pressure recordings were obtained using either the water-perfused HRM system (Solar UGI H20, Trolley system^TM^, Laborie/Medical Measurement Systems, Enschede, Netherlands) or the solid-state high-resolution manometry system (Solar HRM Solid State, Trolley systemTM, Laborie/Medical Measurement Systems). The classification of achalasia into its different types was done based on Chicago classification version 4.0 [2].

Autonomic function testing (AFT)

The subjects reported to the Autonomic Function Laboratory in the afternoon for AFT and heart rate variability analysis (HRV). The study was done two hours after completing the meal. Initially, the patients were familiarised with the study protocol, and then they were requested to rest in a supine position for 10 minutes. ECG electrodes, equipment for continuous beat-to-beat monitoring of blood pressure [Finometer™ (FinometerMIDI™, Finapres Medical Systems, Amsterdam, Netherlands)], and a respiratory belt were attached. Continuous recording of lead II ECG, beat-to-beat blood pressure, and respiratory movements was done using LabChart Pro8.0TM software developed by AD Instruments (Bella Vista, Australia). The acquisition of the beat-to-beat blood pressure, lead II ECG, grip force, and respiration was done. The systolic blood pressure (SBP) and diastolic blood pressure (DBP) were derived from raw blood pressure, while the heart rate and RR interval were derived from the lead II ECG.

The HRV test-ECG was recorded in the supine position for five minutes. The subjects were instructed to close their eyes and to avoid talking and moving. The ECG signal was continuously recorded, amplified, and recorded using LabChart Pro software for further offline analysis. The processing was done using aHRV 13.4.0TM, (NevroKard Kiauata, Izola, Slovenia) HRV software. The R waves were detected and plotted against time to obtain tachograms. Further analysis was done using time domain methods, frequency domain methods, and Poincare plots.

Head-up tilt test (HUT)

In the supine position, the subjects were fastened onto the tilt table (ankle, waist, and chest). Baseline recording of all the parameters was taken for two minutes, and then the table was tilted from 0 to 70 degrees, and the subject was kept at 70 degrees tilt for five minutes. Blood pressure was recorded at baseline, 0.5, 1, 2.5, and 5 minutes. After five minutes, the table was tilted back to 0 degrees, and recovery pressure was noted for two minutes. A fall in systolic blood pressure was noted, and a 30:15 ratio of R-R intervals was noted.

Deep breathing test (DBT)

The test was done on sitting posture. A respiratory belt was placed around the chest at the level of the 5th intercostal space to record respiration simultaneously with the electrocardiogram. Subjects were instructed to breathe deeply and smoothly at 6 cycles per minute, 5 seconds for inspiration, and 5 seconds for expiration. Six such cycles are repeated. The average change in heart rate and expiration/inspiration ratio (E/I), the ratio of the maximum RR interval during expiration to the minimum RR interval during inspiration, was noted.

Valsalva maneuver (VM)

The test was done in a sitting position. The subject was instructed to blow into a mouthpiece attached to a sphygmomanometer. The expiratory pressure was kept at 40 mmHg for 15 seconds. At the end of 15 seconds, the pressure was released. Valsalva ratio (VR), the ratio of the maximum RR interval after the release of pressure to the minimum RR interval during the application of pressure, was noted. Pressure changes during phase II and phase IV, and pressure recovery time (PRT) were noted.

Handgrip test (HGT)

The test was done on sitting posture. Initially, the baseline blood pressure was recorded in the subjects. The subjects were instructed about the test and demonstrated the procedure to use a grip force transducer. After the instruction, the subjects were asked to apply their grip using maximum force with their dominant hands for a few seconds. The value was recorded, and the procedure was repeated three times. The maximum value of the three readings was considered their maximal voluntary contraction (MVC). The subjects were then instructed that they would have to maintain a sustained grip on the grip force transducer at 30% of the MVC for four minutes. The values of the force generated were made to appear on the screen of the computer to aid the patient. Blood pressure was noted at baseline, one, two, and four minutes. One more reading was taken two minutes after the release of the grip.

Cold pressor test (CPT)

First, the baseline BP was taken, and then the subjects were instructed about the test. The cold water was prepared (10 °C), and then the subjects were asked to immerse the hand in water up to the wrist for one minute. Then the hand was removed from the water and covered by a towel. Blood pressure was assessed at baseline, just before the hand was taken out of the water. The blood pressure was taken again at two minutes and four minutes after the hand was withdrawn from the cold water.

Statistical analysis

Statistical tests were done on GraphPad Prism (version 9.0). The results are presented as mean ± standard deviation (SD) for parametric data and median [interquartile range (IQR)] for non-parametric data. The data were tested for normality using the Kolmogorov-Smirnov test, the D’Agostino and Omnibus normality tests, and the Shapiro-Wilk normality test. The comparison of groups was done by the Welch ANOVA test, the Dunnett T3 test, the Kruskal-Wallis ANOVA test, and Dunn’s test.

## Results

A total of 100 patients with clinical and radiological signs suggestive of achalasia were recruited from the Departments of Surgical Discipline and Gastroenterology and Human Nutrition. HRM was performed in all of them and they were diagnosed as different types of achalasia using Chicago classification 4.0. Twelve subjects dropped out (not willing to do AFT and HRV studies); moreover, six patients with EGJ obstruction and 20 patients with other esophageal motility disorders were excluded from the study. AFT and HRV testing were done on 62 patients (30 achalasia type I, 28 type II, and 4 type III). Data of 39 age- and sex-matched healthy controls were obtained from a laboratory historical normative database for comparative data analysis. The mean duration of symptoms; Eckardt score, which assigns points from 0 to 3 for the severity of four disease symptoms (dysphagia, regurgitation, chest pain, and weight loss); and dysphagia for both solids and liquids were highest in type I achalasia, followed by type II and III (Table 1).

**Table 1:** Demographic profile, symptoms, and manometric characteristics of the subjects *P<0.05, type I more than type II achalasia IRP: integrated relaxation pressure; POEM: peroral endoscopic myotomy; SD: standard deviation

Characteristics		Healthy controls	Achalasia type I	Achalasia type II	Achalasia type III
Frequency		39	30	28	4
Age, years, mean ±SD		33.58 ±12.78	32.42 ±12.23	34.80 ±10.44	38.5 ±9.19
Gender	Male	20	18	14	3
	Female	19	11	12	1
Duration of symptoms, years, mean ±SD			6.98 ±4.69*	4.48 ±6.20	0.83 ±0.26
Eckardt symptom score, mean ±SD			8 ±1.92*	6.86 ±1.75	7 ±1.46
Dysphagia (solids/solids+liquids), %			100%, 48.28%	100%, 29%	100%, 0%
Diagnosis post-surgery (Heller’s myotomy, POEM), %			100% absent contractility	80% absent contractility	100% absent contractility
				10% achalasia type 1	
				10% ineffective esophageal motility	
Mean IRP, mmHg, mean ±SD			23.09 ±9.63	27.73 ±18	18.89 ±5.3
Mean IRP post-surgery, mmHg, mean ±SD			5.17 ±2.63	4.57 ±3.67	3.28 ±2.23

Post-surgical intervention, the majority of the patients had findings of absent contractility, and significant improvement of integrated relaxation pressure (IRP) was noted in all types of achalasia patients. However, the requirement for water to assist in swallowing was also noted in all patients. The mean duration of symptoms, Ekardt score, and dysphagia for both solids and liquids were highest for achalasia type I, followed by type II. The mean IRP was found to be higher for type II than for type I. Post-surgery, the mean IRP was found to be higher for achalasia type I than type II, and the majority of patients had 100% failed peristalsis (Table 1).

Tests for parasympathetic reactivity used in the study included DBT, VM, and a 30:15 ratio during HUT. In VM, VR, and on HUT, the 30:15 ratio values of the patients with achalasia were found to be significantly lesser than the healthy controls (shown in Table 2). The parameters of DBT for both types of achalasia were comparable to those of healthy controls. Baroreflex-dependent sympathetic reactivity was tested using HUT and VM, but the findings were comparable to those of healthy controls. There was no significant fall in systolic or diastolic blood pressure in any patient with achalasia, and no recording of orthostatic hypotension was made. During VM, none of the patients with achalasia showed a drop of more than 20 mmHg during phase II. Tests for baroreflex-independent sympathetic reactivity used in the study included HGT and CPT. It was found that in both HGT and CPT, the rise in diastolic pressure for all types of achalasia patients was significantly less than that for healthy controls (Table 2).

**Table 2:** Comparison of parasympathetic reactivity and sympathetic reactivity in different types of achalasia with those in healthy controls *P<0.05, less than healthy controls CPT: cold pressor test; DBP: diastolic blood pressure; DBT: deep breathing test; E:I: expiration:inspiration; HR: heart rate; HUT: head-up tilt; HGT: handgrip test; IQR: interquartile range; SBP: systolic blood pressure; SD: standard deviation; VM: Valsalva maneuver

Test	Parameters	Healthy controls	Achalasia type I	Achalasia type II	Achalasia type III
DBT	∆HR, mean ±SD	24.72 ±7.38	26.07 ±10.74	24.59 ±10.82	24.80 ±10.44
	E:I, mean ±SD	1.42 ±0.14	1.36 ±0.16	1.32 ±0.16	1.35 ±0.17
VM	VM ratio, mean ±SD	1.92 ±0.40	1.54 ±0.28*	1.55 ±0.29*	1.52 ±0.28
HUT	30:15, mean ±SD	1.43 ±0.23	1.15 ±0.13*	1.18 ±0.16*	1.16 ±0.15*
HUT	∆SBP, median (IQR)	0 (0-7)	1 (0-10)	0 (0-4.75)	0 (0-6.25)
HGT	∆DBP, mean ±SD	20.36 ±8.35	15.13 ±8.16*	16.08 ±10.11*	15.54 ±8.45*
CPT	∆DBP, median (IQR)	20 (16-24)	11 (5.5-16)*	11 (7-19.5)*	0 (0-4.75)*

In HRV analysis, a five-minute short-term assessment was done. Among the indices used to quantify parasympathetic influence, all types of achalasia patients had significantly lower values for the standard deviation of differences between adjacent RR intervals (SDSD), the square root of the mean of the sum of the squares of the differences between adjacent RR intervals (RMSSD), and percentage of the number of pairs of adjacent RR intervals differing by more than 50 milliseconds in the entire recording (pNN50) (time domain analysis), power in the low-frequency range of 0.04 to 0.15 Hz (LF), power in the high-frequency range of 0.15 to 0.4 Hz (HF), and total power of variance of all RR intervals (total power) (frequency domain analysis). The median values were lowest for achalasia type I. Among the indices for quantifying sympathetic influence, all types of achalasia patients had significantly lower median values, and the lowest values were seen for achalasia type I. This indicates that though the individual values of LF and HF were significantly lower than the healthy controls, the sympathovagal balance was maintained in all types of achalasia (Table 3).

**Table 3:** Comparison of time domain analysis and frequency domain parameters of HRV between healthy controls and different types of achalasia patients *P<0.05, less than healthy controls HF: power in the high-frequency range of 0.15 to 0.4 Hz; HRV: heart rate variability; IQR: interquartile range; LF: power in the low-frequency range of 0.04 to 0.15 Hz; pNN50 (time domain analysis): percentage of the number of pairs of adjacent RR intervals differing by more than 50 milliseconds in the entire recording; RMSSD: the square root of the mean of the sum of the squares of the differences between adjacent RR intervals; SD: standard deviation SDSD: standard deviation of differences between adjacent RR intervals; total power (frequency domain analysis): total power of variance of all RR intervals

Parameters	Healthy controls	Achalasia type I	Achalasia type II	Achalasia type III
SDSD, ms, median (IQR)	30.69 (25.42-38.16)	20.79 (13.19-35.48)*	23.34 (13.50-33.42)	20.79 (13.19-35.48)*
RMSSD, ms, median (IQR)	33.60 (26.11-55.52)	18.54 (10.00-35.43)*	22.62 (13.35-33.75)*	20.11 (13.08-35.72)*
pNN50, %, median (IQR)	1.26 (0.25-6.05)	0.44 (0.00-2.43)*	1.56 (0.00-5.25) *	0.66 (0.00-4.76)*
LF, ms^2^, median (IQR)	457.7 (368.6-659.7)	121.2 (59.2-230.4)*	154.4 (80.2-343.8)*	170.9 (78.7-400.8)*
HF, ms^2^,median (IQR)	452.5 (292.9-670.0)	92.41 (35.5-294.6)*	156.2 (77.15-314.1)*	141.1 (71.03-375.5)*
LF/HF, mean ±SD	1.21 ±0.32	1.4 ±1.03	1.21 ±0.74	1.37 ±0.90
Total, ms^2^,median (IQR)	1668 (1156-1877)	547.5 (198.9-1113)*	651.7 (324.3-2410)*	649.9 (279.6-1368)*

These findings point to a loss of parasympathetic influence in patients with achalasia. In the parameters indicative of sympathetic influence, such as LF and total power, patients with achalasia type I and II had values significantly lower than those in healthy controls. In the parameters indicative of parasympathetic influence, such as HF and total power, patients with achalasia type I and II had values significantly lower than healthy controls. However, LF and HF showed no significant differences, but sympathovagal balance was maintained. The median values for type I were less than those for type II.

## Discussion

The present study showed that achalasia progresses from achalasia type II to I and may be associated with extraesophageal or systemic alteration of the autonomic nervous system. The study was done to evaluate the autonomic functions in different types of achalasia and compare them with the autonomic functions of age- and sex-matched healthy controls. The Padova hypothesis states that in achalasia cardia, the disease progressed from type III to II and then I [10]. In our study, we also found that the mean duration of symptoms was highest in achalasia type I, followed by type II; the Eckardt score, which quantifies the severity of the disease, was highest for achalasia type I; and the incidence of dysphagia for both solids and liquids was also highest for achalasia type I, followed by type II.

Post-surgery, the majority of patients had 100% failed peristalsis, which is mainly seen in achalasia type I. The results of AFT revealed a generalized loss of parasympathetic and baroreflex-independent sympathetic reactivity in all types of achalasia. However, baroreflex-dependent cardiovascular adrenergic reactivity was normal. In cardiac autonomic tone, there was a loss of parasympathetic and sympathetic influence, but sympathovagal balance was maintained. The severity of the loss of autonomic functions was higher in type I, followed by type II. Autonomic functions were also affected by type III achalasia. As the study was done in a tertiary health care center, the majority of patients recruited were in an advanced stage of disease and had either type I or type II achalasia.

Among the previous studies showing a loss in parasympathetic reactivity, those by von Herbay et al. [11], Olk et al. [12], and Ohlsson et al. [13] showed similar results. The arterial baroreceptor reflex exerts a strong influence on parasympathetic activity [14]. Baroreceptor afferent nerves finally project onto the nucleus ambiguous and DMN [15]. Orthostatic stress evokes a sequence of compensatory cardiovascular sympathetic, parasympathetic, and baroreflex responses to maintain homeostasis [16]. Increases in baroreceptor activity during increases in BP reflexively increase parasympathetic activity, thereby reducing HR. In VM, phase II consists of a fall in BP early in the phase with a subsequent recovery of BP late in the phase.

These BP changes are accompanied by an increase in HR and vagal inactivation. Phase IV of the maneuver is characterized by an increase in BP above the baseline value, leading to vagal activation [17]. In DBT, respiratory sinus arrhythmia occurs due to the modulation of cardiac vagal efferent activity by the central respiratory drive and the gating of excitatory input to the vagal motor neurons by the lung inflation reflex [18]. Both the parasympathetic reactivity tests that showed significant change (VR and 30:15 ratio) are baroreflex-mediated with sympathetic stimulation. However, baroreflex was found to be normal, and hence the pathology could be occurring in the vagal nuclei or the vagal fibers. The cadaveric microscopic study by Cassella et al. [6] reported a loss of ganglion cells within the esophageal myenteric plexus, degenerative changes within the vagus nerve, and a marked reduction of dorsal motor cells within the vagal dorsal motor nuclei in achalasia patients.

Stimuli from exercising muscle, conveyed by lightly myelinated mechanosensitive group III and unmyelinated chemo-sensitive group IV muscle afferents, and the central nervous system (central command) are responsible for the increase in efferent sympathetic activity. The cold water causes stimulation of type C fibers, and the information is carried to the brain through spinothalamic pathways. This in turn would cause an increase in sympathetic stimulation to the heart and vessels [19]. Both the sympathetic reactivity tests that showed significant change (HGT and CPT) are baroreflex-independent. Eckardt et al. [20] also documented similar findings. The pharmacological and histological studies done on isolated LES by Misiewicz et al. [21] found an imbalance between alpha- and beta-adrenergic receptors in achalasia patients.

Sustained changes in cardiovagal nerve activity translate into changes in average (mean) HR, while rhythmic oscillations in cardiovagal nerve activity translate into increases in HRV [22]. In previous studies by von Herbay et al. [11] and Olk et al. [12], RMSSD was found to have been significantly lowered. Rivera et al. (2021) studied the inter-beat interval of heart rate and changes in SBP variability in achalasia patients in supine and standing and controlled breathing at 0.1 Hz and found that there were significant differences in HRV in the time domain and frequency domain parameters. Reduced physical activity, pain, and discomfort due to the morbidities of the diseases could also decrease HRV [8].

The studies by Trudgill et al. [23], Rinaldi et al. [24], and Herreros et al. [25] found that cardiovascular autonomic functions and HRV were not affected. The sample size of the studies was much smaller: the number of achalasia patients included was 19, 19, and 30, respectively. However, in the study by Herreros et al. [25], 11 patients with achalasia and 11 controls were found to have autonomic dysfunction. The contradictory results obtained could also be due to the patchy involvement of the autonomic nervous system. Genetic studies have localized the causative genes in the major histocompatibility complex (MHC) region on chromosome 6, which also has causative genes for diseases like type 1 diabetes mellitus, Sjogren’s syndrome, and systemic lupus erythematosus having systemic or global autonomic dysfunction [26].

Histopathological studies have also shown that achalasia patients had high titers of apoptosis, proinflammatory, and profibrotic cytokines, of which profibrotic and apoptotic cytokines were highest in achalasia type I [27]. Our findings also point in this direction and show that generalized or systemic autonomic dysfunction occurs in achalasia. Currently, the approach to the treatment of achalasia involves providing symptomatic relief and dilatation of the EGJ, but a holistic approach is required. Quantification of autonomic reactivity and tone could have a prognostic value where the severity and stage of the disease could be determined, and this in turn could be used to decide the appropriate treatment modalities. There is also a possibility of autonomic dysfunction in other parts of the gastrointestinal tract and other major organs of the body. Further studies quantifying the levels of autoantibodies, the type of achalasia, and autonomic dysfunction would be helpful in better understanding the pathophysiology of achalasia, the identification of new biomarkers, and potential targets for early identification and treatment of the disease.

Limitations

The number of type III achalasia patients was lower for the comparison of autonomic function changes in different types of achalasia. The inclusion of anatomical (esophageal biopsy) and biochemical (pancreatic polypeptide, antiganglionic antibodies in the blood, etc.) studies might have provided more evidence to prove our hypothesis. Moreover, including gastrointestinal motility studies could have provided further evidence regarding vagal dysfunction. Detailed food diary, microbiota studies, drug history, family history, and quality of life assessment could have provided insights into the epidemiology, prognosis, and treatment of achalasia.

## Conclusions

Based on our findings, achalasia type I is associated with the longest duration of symptoms and the highest Eckardt score, followed by type II and III. Autonomic function tests among achalasia patients revealed a significant decrease in parasympathetic and baroreflex-independent sympathetic responsiveness in all types of achalasia cardia. Furthermore, HRV testing revealed that achalasia patients maintain a sympathovagal balance, but both parasympathetic and sympathetic influences are reduced.
